# Proposed Extension of the Glasgow Maternity Hospital

**Published:** 1904-12-10

**Authors:** 


### PROPOSED EXTENSION OF THE GLASGOW MATERNITY HOSPITAL.

A largely attended and influential meeting was held in the Grand Hotel, Glasgow, last week with the object of bringing the proposed scheme of extension of the Maternity Hospital before the public in order to raise the necessary funds. The Hon. the Lord Provost (Sir John Ure Primrose, Bart., LL.D.) presided. The meeting was under the direct patronage of the Princess Louise, Duchess of Argyll, who was accompanied by the Countess of Eglinton.

After thanking her Royal Highness for her kindness in being present and thus evincing her deep interest in the cause of charity and philanthropy, Sir J. Ure Primrose called upon Mr. Murdoch Cameron, Professor of Midwifery in Glasgow University and one of the consulting physicians to the hospital, to bear testimony to the work done by the hospital. Professor Cameron, speaking from experience gained during more than eight years' work as one of the obstetric physicians to the hospital, spoke very strongly as to the beneficent nature of the work, and dwelt on the urgent need for such an institution in a city where 30 per cent. of the houses are of one apartment and 40 per cent. of two apartments. In the last ten years over 29,000 confinements had been attended, and 90 per cent. of the patients were married women, the wives of poor labouring men. The immense boon to the patients and children of the skill and attention ungrudgingly bestowed can hardly be exaggerated; but, besides this, the institution does invaluable work in training nurses who go forth fully equipped throughout the length and breadth of the land.

The Duke of Argyll moved "That, in view of the urgent need that exists for increased accommodation for patients and for the training of students and ladies' nurses, this meeting resolves to support the directors of the Maternity Hospital in their efforts to effect both these objects!" Speaking on behalf of the Princess as well as himself, he warmly advocated the cause, declaring that it only required to be more widely known to be generally taken up. In the course of his remarks he caused some amusement by laying great stress on the fact that almost all the babies born in the Glasgow Maternity are British citizens and not aliens!

Professor Simpson, of Edinburgh, in seconding, dwelt on the great importance of such hospitals as teaching schools for students and nurses—those nurses who go forth as ministering angels to palace and cottage alike.

Lady Ure Primrose moved "That this meeting resolves to do all in its power to raise the funds necessary for building the required additions to the Maternity Hospital." In a few graceful words she bore eloquent testimony to the service rendered to suffering womanhood, not in Glasgow alone, but throughout the West of Scotland. Dr. Macleod, in seconding, declared that he had never known till now how great a work was being done in their midst, and took the directors to task in characteristic fashion for the excessive modesty which they had displayed.

Thus, in simple, pathetic words that went home to many hearts, he touched on the relief to body and mind alike which such an institution affords to women at the hour of her greatest need. He said it was a Christ-like work, and he said so none the less though a small percentage of the patients were erring sisters. The kindness and sympathy shown to these poor creatures at such a time might be the very means of leading them to repentance.

Robert Gourlay, Esq., LL.D., then moved a vote of thanks to her Royal Highness Princess Louise and to the Chairman, which the Lord Provost duly acknowledged.

Tea was then served to the large company.

The site of the new hospital has been purchased for £14,500. It is quite close to the present building, so the two can be joined by a corridor. The new building will be for maternity cases alone—90 beds—and the present building will be used for gynæcological cases—40 beds. There will be accommodation for 50 probationers in the new building, each with a separate room.

Last year the directors sent Professor Robert Jardine and Dr. Munro Kerr, the two physicians to the hospital, Dr. Macintosh, superintendent of the Western Infirmary, and Mr. Bryden the architect, to the Continent to visit a number of the best maternity hospitals there. The experience gained was utilised in the drawing up of the plans of the new building. It should therefore be up to date in every respect. When the new building is completed, it will be the finest maternity hospital in the Kingdom, and with the wealth of clinical material at hand Glasgow should become a great school for the teaching of obstetrics.

£60,000, of which £15,000 are already in hand, will be required, but in such a wealthy community as Glasgow and the West of Scotland there should be little difficulty in raising the sum.

